# Book Reviews

**Published:** 1811-09

**Authors:** 


### CRITICAL ANALYSIS
### OF
### RECENT PUBLICATIONS
### IN THE
### DIFFERENT BRANCHES OF PHYSIC, SURGERY, AND MEDICAL PHILOSOPHY.

---

*Medical Papers, communicated to the Massachusett's Medical Society.* Published by the Society. Vol 2. Part II. 8vo. Boston. 1810.

We have great pleasure in noticing the progressive state of this Society. The first number of its papers, containing a selection of important communications, was published in 1790. The second, from want of funds, did not appear till 1806. A third, forming the first volume, was printed in 1808 ; the first part of the second volume was published in 1809, which this number completes ; and from the increasing respectability of the Society, a volume may, in future, be expected annually.

The number before us contains only two papers, the last of which we shall notice the first, being *a Dissertation on the Progress of Medical Science, in the Commonwealth of Massachusetts,* read at the annual meeting of the Medical Society at at Boston, June 6th, 1810, by Joseph Bartlett. From this ingenious communication, we learn that little is known respecting the earliest physicians in the state, notwithstanding the establishment of Harvard university in 1638, and the various records and traditions of that period. The first medical publication appeared in 1677, and is entitled, " A Brief Guide in the Small-pox and Measles," by Thomas Thatcher, a Clergyman and Physician. About the same time was published, without the author's name, a Letter concerning a good Management, under the Distemper of the Measles.

The beginning of the next century was memorable for the controversies excited by the introduction of variolous inoculation in Boston, by Cotton Mather, a celebrated divine. Most of the clergy strenuously supported the novel practice.

Amongst the most sturdy opponents were Lawrence Dalhound, a Frenchman, and Dr. Douglas, a native of Scotland; whilst the writing of Dr. Boylston had great influence in establishing inoculation. " His experiments commenced with his son in 1720, and in a year he extended the disease to 247 persons, of whom but six died."

Besides the tracts on measles and small-pox, during a century and a half, the only other medical publication, was a tract on pharmacy, written in 1732, by Thomas Harward, a clergyman.

The first medical establishment was an hospital at Rainsford's island, in the harbour of Boston, which, for upwards of a century, has been appropriated to the reception of mariners and others with " contagious sickness." Hospitals for inoculation were opened in the vicinity of Boston in 1764; and in a few years afterwards, at various other places.

" The first information of physicians in an associated capacity, is in the preface to Douglass, which is addressed to a medical society in Boston; but there are no particulars respecting it. A gentleman lately deceased, whose memory included a retrospect of sixty years, and who knew the author, had no recollection of its existence."

In 1771 an anatomical society existed at the university, and " held private meetings for a discussion of medical and physiological questions, and *were in possession of a skeleton;* but their demonstrations were confined to the dissection of appropriate animals, as the examination of the human body was then an extraordinary occurrence with our most inquisitive anatomists."

" Obstetrick attendance, except in the most difficult cases, was seldom by male practitioners, till within the last sixty years; but this part of the profession is now principally conducted by physicians.

" Though some individuals have been celebrated in particular branches branches of practice, there are no established distinctions, as in other countries; the utility of which has been considered problematic."

The American revolution, and the war consequent upon it, favoured the advance of medical knowledge.

"The establishment of military hospitals afforded extensive opportunities for observations and experiments; important operations in surgery were rendered familiar; whilst the diseases and casualties of camps were constantly occurring. Anatomy was greatly improved by a frequent inspection, without fear of detection, of the organs of the human body; physiology was more accurately comprehended, and a laudable spirit of inquiry was assiduously cultivated."

In 1780, the first course of anatomical lectures in this commonwealth, with dissections and demonstrations, was delivered by John Warren, Surgeon of the hospital at Boston.

The Massachusetts Medical Society was established in 1781, and soon acquired a high degree of consequence in the state; for we find, that in 1789 it "was authorized to point out and describe such a mode of medical instruction, as might be deemed requisite for candidates, previous to examination."

"It was then determined that every pupil should have a competent knowledge of Greek, Latin, the principles of geometry, and experimental philosophy; and that the period of instruction should in no case be less than three years, with attendance on the practice of a respectable physician.* Publications are made triennially of authors to be studied, by which the most valuable modern productions are extensively circulated. The censors meet for examining and licensing candidates once in four months."

In 1803 the constitution of the Society was essentially changed, and its power and privileges extended by an act of the legislature. In 1808, a Pharmacopœia of the Society was published, after the plan of that adopted by the Edinburgh College.

The medical school† at Harvard University, from our author's description, promises to rival the most celebrated in Europe, and has already produced several able physicians.

The improvement of our art is slow and hardly perceptible; but when we reflect on the changes which have occurred in it during the course of the last century, the advance is obvious.

"In May, 1682, notice was given in a London gazette, that as the weather was growing warm, his majesty would not touch any more for

---

* No candidate can be admitted to an examination after June 4, 1813, unless he has studied with, and attended the practice of a fellow or honorary member of the Society. By Laws, p. 18,19.

† An account of this Institution is inserted in the Medical and Philosophical Intelligence of our Journal for the present month.

the the king's evil, till after Michaelmas; and, in 1687, an indigent citizen of New Hampshire, having tried every other means without effect, petitioned the legislature for aid to transport him to England, for that efficacious remedy."

The Massachusetts Humane Society, founded in 1786, was incorporated in 1791, and has 587 members.

Since the year 1799, vaccination has been practised in the state with considerable success, and institutions have been appointed to aid its progress.

Besides editions of ancient and approved medical works, scarcely a publication of importance issues from the British press, without being shortly reprinted, and extensively circulated in America.

We have thus taken a rapid sketch of Dr. Bartlett's interesting dissertation; it has convinced us that the spirit of inquiry which animates our transatlantic friends, will not suffer them to rest satisfied with imbibing their medical principles from European sources; they have now universities, professors, and libraries; enjoy legislative protection, and have perfect freedom from those restrictions and impediments to science, which enacted in a less enlightened age, continue to limit the utility of some of our public institutions.

The article which is placed first in the present number, and to which we now solicit our readers attention, is a *Report respecting a Disease commonly called Spotted or Petechial Fever, which has within a few years been epidemic in various parts of New England.* A Committee* was appointed by the counsellors of the Massachusetts Medical Society, at their special meeting, March 27th, 1810;—

"To frame a series of questions respecting the causes, history, and mode of treatment of the disease now existing in some parts of this commonwealth, commonly called spotted or petechial fever, to address copies of the same to the physicians who reside in places where the disease has prevailed, or does now prevail, and to request the most speedy and minute replies to the same."

The report before us is the result of the exertions of this Committee. The questions framed on the occasion, were highly judicious, and calculated to direct the practitioner in his inquiry concerning the nature of a disease which excited considerable alarm throughout New England. It first appeared in the winter and spring, chiefly in the cold months, in an inland and elevated country, abounding with hills and vallies, ponds, running streams, and fresh water rivers.

---

* The names of the committee are, Thomas Welsh, James Jacks, and J. C. Warren.

"In Cambridge-port, the first place near the sea-coast, at which it was observed, it was confined for the most part to the land which was recently salt meadow, and which is now intersected by many foul ditches. In Boston this disease, as also typhus, has occurred most frequently in those parts of the town exposed to the flats and water."

The disease in a large proportion of cases was mild; in some severe; and in a few destroyed life suddenly like the plague. Whether severe or mild, "the symptoms differed only in degree, not in kind." The communications made to the committee related chiefly to the disease in its gravest forms; from their abstract we select the following particulars of its history.

"The invasion of the disease is generally sudden and violent. In its course all the functions of the body are more or less interrupted, and often some of them are entirely suspended. The subject of it is seized in the midst of his usual labour or occupation, and oftentimes is struck down suddenly, almost as by a stroke of lightning. The first symptoms are various, such as local pain or paralysis, delirium or coma, and rarely spasms or convulsions.

"The disease often commences with shifting pains. The patients suddenly feel a pain in one joint or one limb, often in a finger or toe, in the side, stomach, back, neck, or head. Sometimes the sensation is like the stinging of a bee, frequently it is most excruciating pain, which at once arrests and commands the whole attention. This pain moves from place to place without losing its violence, generally approaching the head, and is often confined to one side of the body. It is said that the left side is more frequently affected than the right. The head is more frequently first affected with pain than any other part; and when not affected at the first moment, it almost invariably becomes so in a short time. The pain in the head is oftentimes intolerably severe, so that it is compared to the beating of hammers upon the part; and the patient says he shall become crazy if it continues.

"Partial loss of sensibility and paralysis are, in other cases, the first symptoms, and often occur in the course of the disease, when they do not in the beginning, The powers of sight are affected in various degrees from a slight dimness to absolute blindness." The sensibility of the skin also is diminished, so that a limb is numbed, "or feels as if it had been asleep,"

"In the muscles of various parts, paralysis has been occasionally observed; as in those of one hand or foot, and oftentimes in those subservient to deglutition. In some cases hemiplegia has occurred at the commencement; and it is particularly worthy of remark, that often the greatest weight of disease falls on one side of the body; insomuch that not only the voluntary muscles, but the vascular system has been much more affected on one side than on the other.

"Not very rarely the disease commences with delirium; and very frequently this symptom follows a violent pain in the head in a very early stage of the disease. The delirium is often mild; in some cases, however, where it is attended with flushed face and eyes, great heat in the head, and violent pulsation of the carotid arteries, it produces a fury fury which is scarcely to be restrained. In a few instances the patient has become blind and raving within half an hour after the attack."

Stupor, coma, convulsions, and spasms, though more frequent in the latter stages, occasionally attend the accession of the disease; and in whatever form it commences, great prostration of strength speedily ensues.

"In some instances the patient is described as almost immediately falling down under the weight of disease. This prostration is accompanied or followed by universal or partial chills; the skin becomes dry and pale, or mottled like one who has been long in the cold; eyes glassy, nose contracted, the face sublivid, with paleness around the mouth, and the countenance expressive of the utmost anxiety and distress, or its features dissolved with a loss of all character and expression; the whole body becomes cold, respiration very laborious, especially in children, pulses very small and feeble, slow at the commencement, but shortly very frequent. If there be neither coma nor delirium, the spirits are very much dejected, the patient suffers extreme solicitude and anxiety, with apprehensions of death, frequent sighs, restlessness, and agitation. He complains of oppression and faintness, with indiscribable distress about the præcordia, and a sensation of fulness at the stomach. Frequently eructation, nausea, and vomiting ensue, and also fainting in the early stages of the disease; and the vomiting occasionally becomes incessant, embarrassing and defeating every effort to give relief by internal medicines, while it exhausts the patients."

The different stages of the disorder have not been accurately distinguished. Diaphoresis usually occurred at an early period, and was followed by a mitigation or subsidence of the symptoms. It is doubtful whether this favourable event was the effect of art, or was a natural termination of the disease.

In general the symptoms became modified in the course of from eight to twenty-four hours; and some patients have died within that period. The second stage may be said to commence, when the pulse becomes more full and regular, the skin warm, countenance flushed, and in plethoric subjects especially, red and florid; respiration short and difficult, but more regular than in the early period of the disease; eye-lids swollen and eyes staring, with a throbbing pain in the head. Light distresses and noise irritates; great restlessness, anxiety, and frequently delirium ensue.

These symptoms usually subside, and the disease terminates within three days; often in one day. Occasionally, after the severity of the disease has abated, it has continued in a milder form, and assumed the character of typhus, in which case, the termination has rarely been fatal.

"Among the varieties of the disease, the following is given as a description of some cases which have occurred especially among females. 'Universal deadly coldness; skin white as polished marble and smooth; countenance perfectly placid ; not one distorted muscle ; pulse in the wrist imperceptible ; motion of the heart scarcely to be felt ; respiration visible only by gasping, and that not frequent ; and as it were only a step between this imperfect state of life and death.' Even from this state of deadly stillness patients have been restored to life and health."

Death rarely occurs after the third day.

"The following is a description of the termination of the disease in cases in which it was fatal within two days. After the symptoms of the second stage, as described above, have continued from six to ten hours, the skin becomes pale and cold ; pulses very quick, small and irregular ; respiration less hurried, but very laborious ; countenance fallen ; the solids flaccid ; and petechial spots of dark colour, violet or livid, suddenly appear on the superior extremities, and immediately over the whole body. At length confusion of mind with constant drowsiness, inability to swallow, respiration more frequent and more laborious, with fluttering pulse, announce the immediate approach of death."

The state of the skin varies considerably ; at the commencement of the disease, it is invariably dry, at a later period sweating has usually occurred with an offensive and peculiar odour. The spots on the skin differed much in different cases. Frequently a rash or miliary eruption only appears, or a few florid or red blotches on the extremities.

"An appearance like measles has also been noticed, and likewise vesicles and pustules, which have been compared to the vaccine and variolous eruptions. In some cases these spots and eruptions have appeared at successive periods two or three times in the course of the disease. The vesicles and pustules are frequently torn by scratching ; after which or without being torn they are commonly followed by scabs of a brown colour ; but occasionally they are followed by ulcerations which do not heal until after recovery. These affections of the skin are often attended with itching ; and independent of them, itching very frequently occurs, especially on the third day, when the symptoms become more favourable."

Petechiæ and vibices occur in but few cases, and indicate danger in proportion as they are dark coloured. In the majority of cases, however, whether fatal or not, there are no spots or eruptions of any kind.

"The tongue is usually moist and white through the whole disease, when it terminates within three or five days. When it continues longer, the tongue becomes darker coloured, yellow or brown. It is sometimes very clean and red"

The thirst is seldom urgent. The appetite is diminished but not entirely lost. Vomiting, but not bilious, frequently occurs. The bowels are quiet and not readily excited to action. The alvine discharge early in the complaint is dark, resembling tar. The urine is scanty, but not altered in appearance.

We

We have now enumerated the leading symptoms of this disease; for those which are occasional, or not of frequent occurrence, we refer to the original publication, from which we have already quoted so largely, that an apology would be requisite, if the work could be readily obtained in this country.

The appearances after death will best indicate the nature of the disease.

" Soon after the patient expires, and in some instances a short time before, the skin assumes a formidable livid colour. This appearance is either generally diffused over the skin, or else it exists in spots, commonly of an irregular form, but occasionally rounded. The lividity is more remarkable at first on the anterior parts of the subject, especially on the fore part of the face, neck, and shoulders, than afterward; for it gradually subsides from these to the posterior parts of the trunk. Whenever the cuticle has been removed by vesication, the skin is almost black and often covered with fluid blood. On the other hand, the petechiæ, which existed during life, become paler, vesicles of phlyctenæ, eruptions and redness of the tunica conjunctiva dissappear."

### HEAD.

" When the cranium is separated from the dura mater, this membrane usually discharges a considerable quantity of blood. As soon as the dura mater is cut through, a quantity of serous fluid commonly escapes from under it. The longitudinal sinus is filled with blood, and when wounded discharges a very great quantity of this fluid, which pours into it from the cerebral veins. Having raised the dura mater, we discover an extraordinary fulness of the veins on the surface of the brain, if the longitudinal sinus is still entire. This appearance, however, varies according to the duration of the disease. In those who have perished within the space of twelve hours from the first invasion, the large blood-vessels are excessively crowded, while, in those of twenty-four hours continuance or longer, the minute vessels are more distinct; and the other appearances we are to describe are more conspicuous in proportion to the duration of the disease. The tunica arachnoides and the pia mater are remarkably altered in appearance, by the effusion of an opaque substance between them, which may be called coagulated lymph, or semi-purulent lymph. This substance is frequently of the yellowish colour of pus, with a consistence between the tenacity of lymph, and the fluidity of pus. At other times we see it possessed of the aspect of well characterized lymph. This effusion accompanies the course of the vessels very generally."—" The two hemispheres of the brain adhere to the dura mater, near the longitudinal sinus, and to each other with so much strength, as to require a laceration or incision through the substance of the brain, in order to arrive at the corpus callosum. The medullary substance exhibits a great number of bloody points at the sections of the vessels, while the cortical part seems paler than usual. The lateral ventricles always contain a notable quantity of water: this varies of course. Sometimes these cavities may be seen greatly enlarged, and at others, with not more than three or four times the quantity often found found in healthy brains. The plexus choroides is often thicker and harder than natural, but always very pale from maceration in the effused water. The membrane attached to the plexus exhibits very considerable alterations from its healthy transparency to a state of morbid thickness and opacity. The membranes at the basis present the same appearances as at the vertex of the brain."

### THORAX.

" The heart generally exhibits some appearance of disease. In every instance the small vessels on the surface of the organ are beautifully injected: the external coat is sometimes the seat of a deposition of lymph, and even the inner lining and valves are occasionally altered from their healthy texture. The right and left cavities usually contain a small quantity of black blood, quite similar in appearance and quantity; and even the aorta has been seen gorged with the same dark-coloured fluid. The structure of the lungs is not commonly deranged."

The abdominal viscera rarely presented any marks of disease. The latest period after death when these subjects were examined " was from twenty to twenty-four hours; at which time there was a less offensive odour exhaled from the body than during life, and there were no signs of the commencement of putrefaction."

The treatment most generally pursued, was to produce early and long continued sweating. The remedies chiefly employed for that purpose were ipecacuanha; and occasionally it was combined with opium, and " *cordials*" were also freely administered. These were aided by external applications, as the warm bath, blankets, hot flannels, &c. This treatment often proved successful, although several practitioners strongly objected to the use of the cordials, which they believed were productive of much injury. In the lethargic state, tincture of opium in large doses was very serviceable; in some cases from fifty to a hundred drops of the tincture administered every half hour " almost invariably removed the lethargy." Arsenic was not much employed, but when used proved beneficial.

" At the same time that cordials have been employed internally, and heat to the general surface of the body, cold water, snow, and ice have been applied to the head. These applications have been made, when there was violent pain in that part with heat and flushed face, and when there was violent delirium. The cold applications have, in these cases, afforded great comfort to the patient, and have mitigated or removed those very important symptoms. Sulphuric æther dropped on the head and allowed to evaporate, has produced similar good effects."

Blisters, when applied to the head or contiguous parts, had great influence in abating the violence of the symptoms; and vesication over the stomach checked the incessant vomiting; mitting, and removed the morbid sensibility of the organ. Cinchona was of little use till the disease was beginning to subside; and, in some instances, preparations of iron were found superior to the bark during the convalescent state. Preparations of quicksilver, especially the submuriate, combined with camphor, ipecacuanha, and opium, and pursued till a slight affection of the salivary glands was produced, were very serviceable, and during the use of these remedies, cordials were not found necessary.

Having recorded the particulars received from their numerous correspondents, the Committee proceeded to make some remarks on the name and character of the disease. The term *spotted* or *petechial* fever has been very properly objected to by most medical men who have considered the subject. The spots, or petechiæ, were not present in a large proportion of cases; when eruption on the skin did occur, the appearance was various in cases where the chief febrile phenomena were very similar; miliary eruptions, blotches, vesicles, and pustules, much oftener occurred than purpura or petechiæ; and the eruptions appeared at uncertain periods of the disease, and were of uncertain duration.

The attempt to solve the question "what is the disease?" is learned and ingenious; from the facts which they have collected and arranged, we think that the Committee have determined correctly, that, *this disease is fever combined with internal inflammation, and the inflammation is commonly erysipelatous.*

The appearances after death clearly established the existence of inflammation of the internal organs, particularly the membranes, and especially those within the cranium. In most instances the inflammation approached nearly in character to the erysipelatous, while in some it corresponded more with the phlegmonous, and was frequently of a character intermediate between the two.

The causes of the disease have not yet been satisfactorily developed.

The treatment pursued by most of the correspondents did not entirely accord with our notions on the subject. The omission of purgatives and bleeding on the accession of the complaint, appeared to us extraordinary. Our apprehensions on the subject, however, were removed by the very rational and, in our opinion, enlightened system laid down by the Committee, with regulations to meet every variety of the complaint; leaving us nothing to add but the testimony of our approbation.

Several cases illustrating the history of the disease are appended.

*A Letter*

*A Letter to Dr. Jones on the Composition of the Eau Medicinale D'Husson.* By JAMES MOORE, Member of the Royal College of Surgeons, Surgeon to the Second Regiment of Guards, and Director of the National Vaccine Establishment. 8vo. pp. 46. Johnson and Co. 1811.

FROM the attention which the Eau Medicinale has attracted in this country, we are persuaded that most of our readers are sufficiently acquainted with the history of its recent importation, and the powerful effects which it has produced in cases of gout. These have claimed additional interest from the class of persons upon whom they have been chiefly induced. It is well known that the nostrum termed Eau Medicinale, besides acting as an opiate, "often proves powerfully emetic and cathartic," and in some cases has operated with considerable violence; persons of exalted rank seldom experience, in these times, such rough treatment from their polite medical attendants, consequently, its beneficial effects were not likely to be duly appreciated. But since the question has been agitated, various ancient authorities in favour of violent evacuations in gout have been adduced. Had these facts been familiar to us, it is not probable, that the effects of the Eau Medicinale on gout would have excited so much astonishment.

From the treatise of Dr. Jones, and from some private experience, we cannot doubt that the nostrum in question frequently alleviates the severest paroxysms of gout, and restores the sufferer to present health, though it does not secure him from future attacks of the complaint. As the composition of the medicine was concealed, various experiments were instituted to ascertain its component parts, but without success. The author of the letter before us, however, has advanced some very strong claims to the honour of the discovery. The mode in which he proceeded was highly ingenious; not trusting to chemistry alone, which had baffled all former inquirers, he called in the aid of metaphysics; or rather by a kind of transmigration into the soul of Mons. Husson, the original inventor, has, possibly, loosed the mysterious knot. Mr. Moore states, that he was encouraged to make the attempt by reflecting, that Husson was, probably, a man of very moderate acquirements, and that consequently "his medicine might be something very obvious, which more learned men might miss by the profoundness of their researches."

From its smell and taste, and its frequently relieving very acute acute pain and promoting sleep, Mr. Moore suspected that opium formed part of the composition of the medicine.

"The point (he continues) was to find out what other ingredients it contained; for, it is evident, that there is at least one possessing qualities very different from those of opium. To detect this, I turned in my thoughts the sensible operations of the medicine on the human body; especially this, that in the small dose of two drams, it often acts with considerable violence as an emetic and purgative, notwithstanding the opium which appears to be in the mixture. The vegetable productions which are known to possess most active powers are few in number; that which suggested itself most frequently to my mind was the root of the white hellebore. This root, it appears by your work, (Dr. Jones's) had also been suspected by a French physician, but on examination was rejected."

Mr. Moore remarks, that the emetic and purgative effects of hellebore were known to the ancient Greek physicians; and Pliny describes it as a most powerful remedy, and enumerates a multitude of diseases which he asserts it cures.—"Medetur (Elleborum album) ita morbis comitialibus, ut diximus, vertigini, melancholicis, insanientibus, lymphaticis, elephantiæ albæ, lepris, tetano, tremulis, *podagricis*, hydropicis, incipientibusque tympanicis, stomachicis, spasticis cynicis, ischiadicis, quartanis quæ aliter non desinant, tussi veteri, inflammationibus, torminibus redeuntibus." Mr. Moore has noticed several passages in Husson's work, which strongly indicate that he took the hint of his medicine from Pliny. Thus in page 24, he observes, "Plusieurs experiences prouvent que l'Eau Medicinale guerit l'epilepsie, la folie accidentelle et recente; elle modère et éloigne les accès de celles invetérées." In another place he writes, "Un des effets les plus extraordinaires de ce remede est la guerison de la folie."

"The above quoted passage from Pliny does not comprehend all the wonderful powers of the white hellebore; he concludes by stating: 'Eodem et Phthiriasis emendatur;' which Husson translates freely by 'Elle a la même empire sur les maladies pediculaires.' Thus, by copying Pliny, he bestows upon the Eau Medicinale a power of curing a disease of which there has hardly been an authentic case, since the death of Sylla."*

---

* Having witnessed cases of this disease, we cannot admit this assertion; from the authority referred to by Bonetus, Sauvages, Willan, &c. &c. the disease would not even appear to be very uncommon.

In his book de Affectibus Capitis, Bonetus describes *Capitis dolor pruriginosus à pediculorum caterva sub pericranio et intra cranii diploas latitante.*

Having related this curious and distressing case, he remarks, "Totum corpus ejusmodi animalculis in prædam cessisse novum non est,

Quis non paveat Pherecydis fata Tragædi?

Sylla quoque infelix tali languore peresus

Corruit, et fœdo se vidit ab agmine vinci.

Upon consulting various writers on the Materia Medica, Mr. Moore found them all agree in asserting that the white Hellebore is a virulent emetic and purgative. It is stated in the New Edinburgh Dispensatory, "that the tincture of White Hellebore is sometimes used for actuating cathartics, &c. "and is an emetic in apoplectic and maniacal disorders." It may likewise be so managed as to prove a powerful alterative and deobstruent in cases, where milder remedies have little effect. But a great deal of caution is requisite in its use: the dose at first ought to be only a few drops; if considerable, it proves violently emetic, or cathartic." Mr. Moore also perceived a striking agreement in taste between the tincture and the Eau Medicinale. From all these circumstances he was inclined to hope that he had discovered the medicine in substance though not in form, and determined to attempt the latter, by making a vinous infusion of Hellebore, and, having filtered it, mixed some of it with tincture of opium. The mixture resembled the taste and appearance of the Eau Medicinale, but had not its peculiar smell. "The root of the White Hellebore is almost inodorous; consequently, the smell of any infusions of that root must depend upon the wine, or the ingredients with which it may be compounded." It then occurred to Mr. M. that Husson being a Frenchman, was likely to adopt some French form; for as no chemical analysis could detect his medicine, the only way was to endeavour to analyse his mind.

"I therefore, (says Mr. M.) examined Les Elemens de Pharmacie, par M. Beaumé, Maitre Apothecaire de Paris, and there found that the Parisian physicians had adopted Sydenham's prescription for their laudanum; which is an infusion of crude opium with saffron, cinnamon, and cloves, in Spanish white wine. I immediately procured a phial of Sydenham's laudanum, and on mixing it with the wine of Hellebore I found that this mixture approached very near to the Eau Medicinale in colour, in taste, and even in smell; and when the mixture had stood for some time, these gradually formed the same cloudy deposit which is so remarkable in Husson's medicine."

The quantity of laudanum Mr. Moore calculated to be one fourth.

We have now stated the principal facts contained in Mr.

Recentissimum vero exemplum suppeditavit annus 1673, exeunte hyeme: E sexcentis Equitibus, agmen generosis. Marchionis de Lussinge Allobrogis, castra maximi Regum sequuti, componentibus, vix decima pars superstes evasit, plerique non armis, cecidere sed dysenteria: Reduces vel pedum gangræna confecit, à diutina in paludoso solo mora, indesinentique ocrearum gestatione; vel *Phthiriasis*: Adeo ingens pediculorum numerus corpus occupabat, ut paucis diebus fœde et misere illi interirent: Ne ipsis thoracibus bubalinis (familiari Equitum vestimento) pepercerunt, in quibus domicilium quæsierunt, crebrisque effossis, cuniculis perterebrarunt et eroserunt."

Moore's

Moore's interesting letter, from which we think that he has at least established an evident resemblance in appearance, as well as in qualities, between the two medicines. Actual experiments upon patients can alone confirm his very probable conjectures, and reasoning upon the subject. Only four cases are stated in the present publication, and in these "the effects of the mixt infusions were precisely the same with equal doses of the Eau Medicinale. In two of the cases where two drams were given, vomiting and purging were produced; and in one case the medicine occasioned constipation, which happens also with the Eau Medicinale; and the gout in all was relieved." Since this letter was published, we hear that benefit has been obtained by the new remedy in several other cases of gout.

To enable our readers to make trial of the medicine, we subjoin the formula as directed by Mr. Moore, and shall hope shortly to receive more information on this truly important subject.

"Take of white hellebore root, eight ounces; white wine, two pints and a half. The root is to be cut in thin slices, and infused for ten days, occasionally shaking the bottle. Let the infusion be then filtered through paper.—The mixture employed for the gout, consisted of three parts of the above wine of white hellebore, and one part of liquid laudanum.

We presume, the dose should be from one to two drams of the mixture, according to the nature of the case, or the operation of the medicine, which varies in different constitutions.

---

*Practical Observations on the Diseases of the Inner Corner of the Human Eye; comprizing the Epiphora, the Tumor Sacculi Lachrymalis, and the Fistula Lachrymalis; with a New Arrangment and Method of Cure. Also, Remarks on Mr. Ware's and Professor Scarpa's methods of treating these Disorders. By* JOSEPH READE, M.D. &c. &c. &c. 8vo. London, 1811, pp. 105. Underwood.

THE object of this Treatise, is to describe the diseases of the inner canthus of the human eye, and to institute, what the author conceives to be, a better method of treatment. Our knowledge of the *Epiphora*, *Tumor Sacculi Lachrymalis*, and *Fistula Lachrymalis*, are confused and obscure, Dr. Reade asserts, in even the best authors. To shew the correctness of this opinion, he gives in his preface an abridged history of the methods of treating the *Fistula lachrymalis* from *Anel* to the present time.

 In

In 1712 Anel being called to attend on the Duke of Savoy, avoided the rude and coarse method of treating Fistula Lachrymalis by cutting, boring, and burning; and introduced a slender probe into the *punctum lachrymalis*, and from thence into the sac, and through the nasal duct into the nose. The obstruction being assumed thus to be removed, he proposed to maintain the opening by injecting tepid water night and morning, or oftener, by an ingeniously contrived syringe; and repeated the introduction of the probe as often as necessary, or until the injection passed freely to the nose, and no purulent matter was discharged either spontaneously, or by pressure, from the lachrymal sac through the puncta. The apparent simplicity of this operation, its occasioning no destruction of parts, and its consonance to nature, gained it, for a time, many partizans. Fantoni, a physician of Turin, Mangetus, Molinetti, Lancisi, Vallisnieri, Morgagni, and Heister, were its panegyrists: but it was very early opposed by F. Signorotti, who published a work expressly against it*; and our countryman Mr. Pott observed, "the passing of a small probe through the puncta has a plausible appearance, but will, upon trial, be found very unequal to the task assigned: the very small size of it, its necessary flexibility, and the very little resistance it is capable of making, are manifest deficiences in the instrument; the quick sensation in the lining of the sac and duct, and its diseased state, are great objections on the side of the parts, supposing that it was capable of answering any valuable end, which it most certainly is not."

Inadequate as the method of Anel was, it had the merit of being founded on a knowledge of the structure of the parts, and in this view led to an improved practice. It was now understood that the disease in the lachrymal sac was occasioned by an obstruction in the *ductus ad nasum*, and all subsequent efforts were directed to restore that passage to its natural state, or to supply it by an artificial opening. To re-establish the natural passage, *Mejan* introduced a seton from the nose into the nasal duct, by means of a needle armed with

* Those of our readers who may have the curiosity to search into the history of an obsolete, though perhaps, not totally useless practice, we refer to "Observation singuliere sur la fistule lachrymale, dans laquelle on appendra la methode de la guérir radicalement. 4to. Turin 1713. Nouvelle méthode de guérir les fistules lachrymales. 4to. Turin. 1713. Suite de la nouvelle méthode de guérier la fistule lachrymale. 4to. Turin. 1714." And a "Dissertation sur la nouvelle découverte de l'hydropisie du conduit lachrymal. 12mo. Paris. 1716."

a silk a silk thread passed from the punctum into the cavity of the nostril. The difficulty of getting the seton into the duct by this means, the irritation it produced when it was drawn in, as well as that occasioned by the silk thread constantly hanging from the punctum, were insuperable objections to Mejan's project. *Jurine*, an oculist of Geneva, attempted to remove the difficulties which arose in the application of the seton, by pushing a trocar into the sac and so on to the nose: through the canula of this trocar he passed the seton. The failures of Anel and Mejan were followed by various methods of treating this disease of the inner canthus, all of which were now founded on a knowledge of the structure and functions of the parts: and their principle was either to restore the natural duct, and where that was impracticable, to make an opening into the nose, through the *os unguis*. From *Laforet* to *Pott* these are too well known to require recapitulation. M. Sebatier details them with much perspicuity; but of his history it must be remarked, that it is totally silent on the elucidation the subject has received from English Surgeons, especially Mr. Pott. This must be referred to one or other of the following causes. M. Sabatier was influenced by nationality, or he was ignorant of what had been done in this country. We are unwilling to load him with the disgrace of the first, and are induced to adopt the latter, because the only Englishman he mentions is *Woolhouse*, a person better known at Paris than in London.*

---

* His obscurity with regard to England, and the rareness of his works in London, may render some short notices of J. T. Woolhouse, not quite unacceptable. He was born in London, was occulist to William the Third, but resided the greater part of his life at Paris. He had a warm dispute with Heister on the nature of cataract, and controverted the opinions of Brisseau, Antoine, Coward, Winslow, St. Yves, and Morand. His publications are, 1. *Experiences de differentes operations manuelles et des guerisons qu'il a pratiquées aux yeux. Paris*, 1711. This was translated into Latin with the title of "2. *Quadraginta circiter operationes Chirurgicæ, quas oculis laborantibus administrat, docetque in Collegio vulgô dictô de l'Ave Maria, juxtà Ecclesiam Parochialem Sancti Stephani de Monte, in Universitate Paresiensi.* 8vo. *Francuff.* 1719."

3. "*Dissertations sur la Cataracte et de Glaucome de quelques modernes et principalement de M. M. Brisseau, Antoine & Heister.*" 8vo. *Offenbach*, 1717. This was translated into Latin by Christopher le Cerf, and published at Francfort, in 8vo. 1719, with the title of "*Dissertationes de Cataracta et Glaucomate contra systema Brissæi*," &c. &c. &c.

"*Catalogue d'instrumens pour les operations des Yeux.*" 8vo. *Paris.* 1696.

"*Observations critiques sur un Livre imprime en Angleterre.*" 8vo. London. 1713.

It

It will be understood from the preceding remarks, that Dr. Reade treats of the diseases of the inner canthus of the eye under the three distinct states or gradations of *Epiphora*, *TumorSacculi Lachrymalis*, *and Fistula Lachrymalis.* These he "nosologically defines" in the following manner:

"*An Epiphora* is a flow of tears, mixed with either pus or mucus, coming from the surface of the eye and falling over the cheek, or pressed by the finger through the *puncta lachrymalia*, without any manifest distinction or relaxation of the sac, and might be divided into two stages, the simple Epiphora, commonly pellucid, composed of tears and lymph; and the muco-purulent Epiphora, opaque and yellow, composed of tears, mucus, and pus.

"*The Tumor Sacculi Lachrymalis*, generally occasioned by the Epiphora, or a lodgment of tears and mucus, sometimes mixed with pus in the lachrymal sac, by which it is evidently distended and relaxed."

"The *Fistula Lachrymalis*, when from an over accumulation of the contents of the sac, inflammation takes place, and the sac bursts, forming a fistulous opening, through which tears, mucus, and pus, are constantly evacuated, sometimes accompanied with a caries of the Os unguis."

In the chirurgical treatment of these diseases of the lachrymal appendages, or rather the different stages of the same disease, the novel method promised by Dr. Reade applies to the *Tumor Sacculi Lchrymalis.* It consists in maintaining a permanent opening at the upper part of the tumid, inflamed, and relaxed lachrymal sac, through which the tears, morbid mucus, or pus accumulated in that sac, are to be frequently discharged by pressure. The history of the author's own case will explain both the principle and practice, and present to our readers a specimen of the style of his pamphlet.

"About ten years ago," he says, "I was attacked whilst at College, with a severe ophthalmia, which, after some time, yielded to the usual remedies; but by degrees a distention and relaxation of the lachrymal sac took place, preceded and accompanied by an Epiphora, soft and easily yielding. Whenever occasion required I pressed out a limpid tear, partly through the puncta, and partly through the nasal canal: however, when I neglected this for a few days, the tears instead of being limpid as usual, became inspissated, and put on the appearance of purulent matter, similar to the discharge in catarrh; the palpebræ and meihomian glands were certainly inflamed from the commencement, not only of the tumefaction, but of the Epiphora. In this stage of the complaint I consulted Dr. Monro, sen. and several other surgeons of eminence in Edinburgh, who were of Mr. Benj. Bell's opinion, that whilst I found what they called little inconvenience or pain from pressing out the contents of the sac, I should not submit to any operation: for some years these symptoms remained stationary, but I must say very disagreeable. At length I experienced greater difficulty and some pain in pressing out the fluid, the sac became more distended and hard. Having read Mr. Ware's treatise, I determined to put myself under his care, and in May

1808,

1808, after having consulted some Dublin surgeons, proceeded to London. Before I arrived the puncta, ducts, and sac, became inflamed and considerably tumified; in some days, however, those symptoms having subsided, I applied to Mr. Ware, who having endeavoured, without success, to inject some warm water into the nose, made an incision into the sac with a spear-pointed lancet, and having discharged the thickened tears and mucus, he introduced a probe into the duct with very little difficulty, and then inserted a nail-headed cylindrical stile, about an inch and a quarter in length, through the nasal duct into the nose; in a few days I was much pleased to find little inconvenience and no pain, being able to withdraw and replace the stile at pleasure. After a week, during which Mr. Ware every second day injected warm water for the purpose of keeping it clear, I returned to Cork, and remained perfectly well for some months, at the end of which time I found great inconvenience and difficulty in introducing the instrument, particularly where it enters through the bone at the bottom of the sac. It occurred to me that this obstruction might be removed by substituting a conical stile, the small end easily passing, the increasing diameter would preserve the duct in a permeable state, and facilitate the introduction. I found this metallic cone to answer the purpose so well, that in operating I have since used it from the commencement; for although slight the improvement might appear at first sight, it oftentimes obviated the necessity of a second operation, especially to those patients remote from surgical assistance, who, after much uneasiness of mind, and inflammation of the parts, from repeated, but nugatory trials, were unable to accomplish the introduction. Having worn this conical silver stile for a year, the parts being perfectly healthy, I determined to discontinue it, my mind cheered with the confidence of a perfect and permanent cure. For one short month all went on in unison with my most sanguine expectations, the tears passed freely into the nose, and I wrote to Mr. Ware a letter of thanks; but in some time I became very uneasy at finding the angular tumor again forming, and that I was obliged to press out the tears through the puncta as formerly: thus from my own case, and several others on whom I operated, and who had again applied to me to relieve returning symptoms, I was concerned to be obliged to form the opinion already stated, that like all others, Mr. Ware's operation is no more than a palliative remedy, and gives relief only as long as the stile is worn.

" At this particular crisis a young lady applied to me to have her ears bored, who five years before had undergone a similar operation, but since that time had not had rings introduced. I was not a little surprised on examination to find the foramina perfectly open, without the least discharge or inflammation; from this circumstance a thought occurred, that a similar orifice might be made in a superior part of the relaxed lachrymal sac, through which the tears might be pressed *ad libitum*, the sac converted into a reservoir, a weeping eye and the deformity of a silver headed stile prevented. Actuated by this opinion I opened the sac of my right eye, at that time very much enlarged and tense, before a looking-glass with a sharp spear-pointed lancet, and introduced a bit of leaden wire, nearly the diameter of a small pin, and about half an inch in length, bent at the top, which remained out of the orifice to prevent it from slipping into the duct. This I wore for a few weeks until all inflammation flammation subsided, and the orifice became fistulous; I then withdrew the leaden wire, and to prevent the orifice from closing, gently pressed out the tears every three or four hours; I say gently, least pressure might induce inflammation and consequent adhesion. At present it is more than a year since I adopted this plan, and now have seldom occasion to squeeze out the tears more than two or three times a day; from this almost imperceptible orifice, the *ductus ad nasum* is perfectly free, as I have as copious a discharge from the corresponding nostril as from the other, and my eyes are remarkably acute and strong, although I am in the habit of nocturnal study.

"Since I discovered this method of cure, I operated in a similar manner on several, in all of whom it has succeeded, from which I think I am authorized to draw the following conclusions.

1st. "That the Tumor Sacculi Lachrymalis is only to be relieved by an operation, and that Mr. Ware's is, at best, but a palliative one.

2d. "That the disease was neither occasioned, at least in this case and many others, by inflammation and a purulent discharge from the palpebræ and meibomian glands, as Scarpa asserts, nor from any primary obstruction in the nasal duct, but arose from atony and distention of the sac, which being thus deprived of its healthy contractile power, allowed the tears to be collected and inspissated, thereby producing a mechanical obstruction. Nevertheless, I by no means mean to say, the disease may not, in some instances, be occasioned by other obstructions in this duct.

3d "That a conical is preferable to a cylindrical stile, and that a small and almost imperceptible orifice in the superior part of the sac is better than either, producing no deformity, being less troublesome than a nail-headed stile placed in the corner of the eye, which is constantly liable either to fall out of the orifice, if too free, or to excite inflammation by the introduction, if too confined; and finally, that when the sac is thus made a reservoir, by placing the orifice at the top, pressing out the tears two or three times a day is as little troublesome as blowing the nose."

Authors have often made that complex and obscure, which in nature is simple and clear. Epiphora, Tumor Sacculi Lachrymalis, and Fistula Lachrymalis, are but symptoms of a disease which has not received a technical appellation. By an accumulation of inspissated mucus obstructing, or by inflammation contracting the *ductus ad nasum*, of ten we believe, in the latter case, occasioned by Erysipelas about the face, and by Small-pox, the exit of the tears from the eye is denied by the natural passage. The first and direct obvious effect is an accumulation of the lachrymal fluid in the eye, until it passes over the lid and runs down the cheek. This is Epiphora, a symptom* of obstruction in the nasal duct. After a longer

* Epiphora, not symptomatic of the obstructed state of the *ductus ad nasum*, may exist, and is connected more simply with a want of balance between the action of the lachrymal gland, and the apparatus for carrying or a shorter time, influenced by attempts injudiciously made to remove the complaint, by accidents, or by idiosyncrasy, the *saccus lachrymalis* becomes distended with accumulated tears, now opake in that portion of them collected in the sac, and approaching to purulency in appearance, being mixed with the diseased mucus of the parts. Pressure on the sac, at this period, will discharge its altered and apparently purulent contents, at the *puncta lachrymalia.* This constitutes the disease denominated *Tumor sacculi lachrymalis*; another symptom of the obstructed or diseased state of the nasal duct. At an indeterminate period, influenced also by the circumstances above stated, the distended *sacculus lachrymalis* inflames, ulcerates, and opens externally. This is the famed *Fistula lachrymalis*, for which, when the structure and functions of the part were not understood, the cautery, both potential and actual, was employed. The bones were injured often by this process; exfoliation followed caries, the delicate *os unguis* was destroyed, and extensive opening into the nose ensued, which maintained itself, and the disease was sometimes cured.

The obstructed state of the *ductus ad nasum* is the original complaint, and the cause of all the other morbid appearances, which exist but as evidence of that obstruction. They are removed by restoring the natural passage into the nose, or by making a new one through the *os unguis*. Modern Surgery very well supplies the means, and Dr. Reade's book may be

---

ing off the tears which have washed the eye. Thus, certain states of mental feeling always; the application of cold sometimes by constricting the puncta lachrymalia, perhaps; and inflammation of the eye, by diminishing the capacity, or closing the puncta, produce watery eye. It is plain that this is the disease that Prof. Scarpa describes as proceeding from a purulent discharge from the palpebræ and meibomian glands. It will be seen that this variety of Epiphora will not produce that state of the sac which has been denominated Fistula lachrymalis. In this variety of Epiphora the tears cannot get into the lachrymal sac; in that variety of Epiphora connected with Fistula lachrymalis, the tears are confined to the sac by obstructions in the nasal duct. When the obstruction is in the ductus ad nasum, disease in the sac ensues; when the obstruction is in the puncta or duct leading to the sac, that cavity is secure. The pathognomonic mark will be the absence or presence of accumulation in the sac. If on pressure applied to the sac, there is no regurgitation of the fluid by the puncta, the conclusion will be, that the obstruction is above the sac: if regurgitation by the puncta follows pressure on the sac, the conclusion will as necessarily be, that the obstruction is below the sac, and in the ductus ad nasum.

 usefully usefully perused with this view. We cannot, however, admit with Dr. Reade, that the method by restoring the natural passage or by making a new one, is only palliative. If a radical cure ever occurs, this is the method by which it must be effected. But Dr. Reade's proposition is in its nature palliative only. As a dernier resource it may sometimes be convenient, but thus far and no farther can it go.

---

*Report of the National Vaccine Establishment.* 8vo. London. 1811. pp. 20.

The Board of the National Vaccine Establishment must necessarily take a lively interest in whatever promotes or retards the progress of Vaccination, and in whatever tends to elucidate the actual properties and powers of that practice. With these feelings, they could not look with indifference on the occurrence of Small-pox after what had been deemed successful Vaccination. Instances of this kind have recently happened in London, and the Establishment, with a candour and liberality that decidedly shews its disposition to ascertain and promulgate truth, has published a detail of two cases, which have come immediately under the observation of several of its members. These happened in the families of the Earl of Grosvenor, and Sir Henry Martin. The particulars of the case of the Hon. Robert Grosvenor, third son of the Earl of Grosvenor, are given by Sir Henry Halford, and Sir Walter Farquhar, who attended him, and by James Moore, esq. Director of the Vaccine Establishment, who occasionally visited the patient during the progress of the disease.

"On Sunday, May 26, 1811, the Hon. Robert Grosvenor, who was recovering from the Hooping Cough, became much indisposed and threw up his dinner. Fever followed, and he complained most particularly of excruciating pain in his back. He dwelt on this symptom until Thursday, when he became delirious, and there were observed on his face about twenty spots.

He had been vaccinated by Dr. Jenner, in his infancy, about ten years ago, and the mark left in his arm indicated a perfect disease.

On Friday morning, the eruption had not increased materially in point of number, but the appearance of the spots and the previous symptoms, suggested strongly a suspicion that the disorder was the Small Pox.

"Sir H. Halford had occasion to go to Windsor in the afternoon of Friday, and did not see Mr. Robert Grosvenor until the Monday following, (June 2d) but he learned from Sir W. Farquhar, who attended him most carefully during Sir Henry's absence, (and subsequently) that the eruption had increased prodigiously in the course of Friday; that that on the evening of that day Mr. Robert Grosvenor began to make bloody water, and that he continued to do so until Monday morning.

"On the tenth day of the disease, the pustules began to dry upon the face, which was swollen to a considerable degree, but not to the extent of closing his eyes, and was attended by a salivation which lasted several days. Petechiæ had occurred in the interstices of several of the spots, particularly on the limbs, and there was that particular smell from the whole frame which is remarkable in bad Cases of confluent Small Pox.

"It was obvious that the first symptoms of which Mr. Grosvenor complained, were such as indicated a violent disease about to follow, and Sir Henry confesses that he entertained a most unfavourable opinion of the issue of such a malady, when it was fully formed: having never seen an instance of recovery under so heavy an eruption attended by such circumstances. It seemed however that the latter stages of the disease were passed through more rapidly in this case than usual, and it may be a question whether this extraordinary circumstance, as well as the ultimate recovery of Mr. Grosvenor, were not influenced by previous Vaccination."

During the illness of Mr. Grosvenor, the other children of the Earl, who had formerly been vaccinated, were exposed to the contagion of the Small-pox, under which their brother was suffering, and were also submitted to Small-pox Inoculation without effect.

The Case of Mr. Martin is given by Dr. Heberden, who attended him with Mr. Tegart of Pall-Mall. In the preceding pages of this number of our Journal Mr. Tegart has given, with much precision, the progress of this case from its commencement to its termination.

It is admitted by the Board that the case of the Hon. R. Grosvenor was a case of confluent Small Pox. That the attack and progress of the disorder were attended with symptoms which almost invariably announce a fatal termination.

"But they observe, that the swelling of the face which is generally so excessive as to close the eyes, and is considered as a favourable symptom, was slighter than usual, that on the tenth day the pustules began to dry upon the face, and that from that time the disease passed with unusual rapidity through the period, when life is generally esteemed to be in the greatest hazard

"Those who are acquainted with the nature of the confluent Small-pox, are aware that this peculiarity cannot be attributed to the effect of medical treatment.

"The case of the son of Sir Henry Martin exhibits a mild form of distinct Small Pox, occurring after Vaccination.

"In most cases of Small Pox which have succeeded to Vaccination the pustules have been observed to dry more rapidly, and the disorder has concluded at an earlier period than usual.

"If allowance be made for the relative periods in which the confluent and distinet Small-pox complete their course, the rapid progress towards recovery recovery through the latter stage of confluent Small-pox, as exhibited in the case of Mr. Grosvenor, may be compared with the rapid desiccation of the pustules in the distinct and peculiarly mild form of the disorder which is considered as Small Pox modified by Vaccination. Both forms of the disorder proceed in the usual course, the one attended with violent, the other with mild symptoms, till they arrive near to the height, when they appear to receive a check, and the recovery is unusually rapid.

"From this correspondence of circumstances, the Board are induced to infer that in the case of Mr. Grosvenor which has been more violent than any yet submitted to them, the progress of the disease through its latter stage, and the consequent abatement of symptoms, were influenced by an antivariolous effect, produced upon the constitution by the Vaccine process.

"The occurrence of Small Pox after Vaccination has been foreseen and pointed out in the Report on Vaccination made to Parliament, by the College of Physicians in the Year 1807, to which the Board are desirous of calling the attention of the Public; wherein it is stated that,

"'The security derived from Vaccination against the Small Pox, if not absolutely perfect, is as nearly so as can perhaps be expected from any human discovery, for amongst several hundred thousand cases with the results of which the College have been made acquainted, the number of alleged failures has been surprisingly small, so much so as to form certainly no reasonable objection to the general adoption of Vaccination; for it appears that there are not nearly so many failures in a given number of Vaccinated persons, as there are deaths in an equal number of persons inoculated for the Small-pox. Nothing can more clearly demonstrate the superiority of Vaccination over the Inoculation of the Small-pox than this consideration; and it is a most important fact, which has been confirmed in the course of this inquiry, that in almost every case in which the Small-pox has succeeded Vaccination, whether by Inoculation or by casual infection, the disease has varied much from its ordinary course; it has neither been the same in violence nor in the duration of its symptoms, but has, with very few exceptions, been remarkably mild, as if the Small-pox had been deprived by the previous Vaccine disease of its usual malignity.'"

With the preceding instances of Small-pox after Vaccination, four cases of that disease after variolous Inoculation and the casual disease came before the public. These were the cases of the Rev. Joshua Rowley, Miss Booth, John Godwin, and Peter Sylvester.

*Mr. Rowley* was inoculated in 1770, by the late Mr. Adair, when he had a considerable eruption of pustules. He was again attacked with the disease on the 5th of June last. The eruption was distinct but numerous: about 200 pustules in the face, and a proportionable number on the trunk and limbs. The statement is from Mr. Guy of Chichester.

*Miss Sarah Booth* of Covent-garden Theatre, was inoculated when five years old, had the disease in a satisfactory manner, manner, was seized with fever on the 20th of June last, which proved the precursor of an eruption of distinct Small-pox. The disease went regularly through its stages, was accompanied with sore throat, and much general disorder.

*Peter Sylvester* was inoculated by Mr. Ring in 1799, and had the disease in a most perfect manner. On the 24th of June last this boy was seized with fever, and on the 27th the variolous eruption appeared. It proved a distinct but severe case of Small-pox.

*John Godwin*, born in 1800, had the Small-pox at six weeks old, and has undergone the test of Small-pox inoculation, without taking the disease. A few weeks since at the age of eleven, this boy had casual Small-pox, from which a child was inoculated, and had distinct variolous eruption.

These accidents, occurring nearly at the same period of time, have led to a comparison of the security afforded by Small-pox Inoculation and Vaccination, not disadvantageous to the latter. On this subject the Report remarks ;—

"The peculiarities of certain constitutions with regard to eruptive fevers form a curious subject for Medical History. Some individuals have been more than once affected with scarlet fever and measles, others have been through life exposed to the contagion of these diseases without effect ; many have resisted the Inoculation and contagion of Small Pox for several years, and have afterwards become susceptible of the disorder, and some have been twice affected with Small-pox.

"Among such infinite varieties of temperament it will not appear extraordinary, that Vaccination though so generally successful should sometimes fail of rendering the human constitution unsusceptible of Small-pox, especially since it has been found that in several instances Small-pox has occurred to individuals over whom the Small pox Inoculation had appeared to have produced its full influence."

The varieties of temperament or idiosyncracies here noticed, are very properly said "to form a curious subject of Medical History." With regard to the influence of temperament over the action of variolous contagion, the history is not only curious but important. Peculiarity of idiosyncracy has given rise to anamolies which the ignorant and the prejudiced formerly opposed to *Inoculation:* and in the present enlightened time, we see these anomalies produced as arguments against VACCINATION. Before the promulgation of Dr. Jenner's discovery, in the course of a moderately extensive practice in the casual and inoculated Small-pox, it had happened to us to observe three conditions of the human frame in which Small-pox was either stopped in, or deviated from, its accustomed course. These conditions were of opposite properties : they consisted of permanent insensibility to variolous Contagion, of temporary rary insensibility, and of unusual aptitude to receive infection.

We have seen an extraordinary insensibility to the contagion of Small-pox exist permanently, in the eldest sons of one family, and have strong evidence to prove that this peculiarity acted for three generations. That is, the grandfather, we trace no further back, being an eldest son, was insusceptible of variolous Contagion all his days, while the brothers and sisters were, with regard to this disease, as others are. The eldest son of this person was also exempted from the effects of this contagion; but his brothers and sisters were not. The eldest grandson enjoyed the same privileges, but his brothers and sisters all took the disease, either casually, or by inoculation. During the period in which we had an opportunity of observing this family, or hearing its history from the sober and sedate individuals of it, we inoculated, at distant intervals, the last of these privileged persons, four times, without any impression being made on his constitutional invulnerability.

We have seen this insensibility to Small-pox contagion exist for a time only. T. C. a respectable farmer, was inoculated for Small-pox about 1790, with others in the same family. A pustule arose on his arm in the place of the puncture, but continued too short a period, and was unaccompanied with any general derangement or febrile state. He slept with those who had the eruption upon them, but did not take the disease. He was considered to be secure. For twelve years he was occasionally exposed to infection, never avoiding those in the disease. At the end of this period he was infected, the case was confluent, his eyes were closed ten days, and his life was put into great hazard.

The following instances of unusual susceptibility to the action of the contagion of Small-pox, both casual and inoculated, we know are perfectly correct.

The mother of P. F. had a severe case of Small-pox when this female child, at six months old, was at her breast. The child had the mother's disease in its severest form, was much marked on the face, and lost an eye.

Nineteen years after this we attended the younger branches of this family, some in the casual disease and others under inoculation, to whom this person, P. F., then between nineteen and twenty years of age, became nurse. During the progress of these cases P. F. sickened with fever, the pustular eruption appeared on the third day, the crop was large on the face and over the surface generally: she was confined to her bed, the face swelled, and the remaining eye was closed for some days.

If

If this peculiar idiosyncracy which renders a person liable to receive the Small-pox a second time, even in the casual or as it has been called natural Small-pox exists, we shall not be surprized to find some instances of insecure inoculated Small-pox, from the same constitutional susceptibility to renew the disease. In the course of our professional avocations, we have inoculated upwards of 6000 persons for Small-pox. Among these we found the individuals of one family, irregularly susceptible of the disease. The name of this family was Taylor, and in the daughters only did we perceive this peculiarity. Fourteen years before the time of which we speak, this family was inoculated: they all had the disease with a severity unusual in the inoculated Small-pox. The subjects of these remarks, two daughters, had the disease to a degree that marked their faces, and indelible vestigia of the operation remained in their arms. The accession of younger branches, and the appearance of Small-pox in the village where they resided, brought the family again under inoculation. These young women then became nurses to their junior brothers and sisters, and both again received the disease; not in the slight and evanescent form in which it has been observed to occur to nurses, but with active fever, a plentiful crop of pustules, and tumor of the face, though not to absolute closing of the eyes.

We could relate a great number of cases wherein Small-pox, either casual or inoculated, had, within our own knowledge, succeeded to former inoculations. But we consider these to have arisen, generally, from negligent or improper employment of the infecting material; and that the disease not having taken place in the first instance, these persons were left the same as if no inoculation had been attempted. In the histories we have given, the facts are marked with a distinctness that shews the actual existence of idiosyncracies, which would if they were sufficiently general, abrogate the established and well understood laws of this disease.

The cases of permanent insensibility to variolous contagion are applicable to vaccination no further, than as they shew the existence of a peculiarity of frame not subjected to the general laws which are known to govern the morbid actions in variola.

The case shewing the existence of a temporary insensibility to variolous contagion, where the person was inoculated while this peculiarity continued, and resisted then and for twelve years afterwards its impression, and at length took the disease in its severest form, applies, by no forced analogy, to the phenomena of Vaccination. If an insensibility to the action of variolous contagion exists for a time, and then subsides, sides, it may surely be allowed that the comparatively mild fluid of the vaccine vesicle may, at one period of time, be resisted by a similar idiosyncracy, which afterward subsiding may leave the system highly sensible to that, or to Small-pox contagion. Had the case of Mr. C—— occurred in the early period of Small-pox inoculation, it would have been used to shew the insecurity of that practice.

The cases of increased sensibility to variolous contagion, subjecting certain persons to have that disease twice, indiscriminately from either casual or inoculated Small-pox, will shew, very plainly, the fallacy of inferring the insecurity of Vaccination from solitary instances of Variola occurring after that practice.

It will appear from the preceding statement, the circumstances of which have fallen under our particular cognizance, that one person in three thousand has received the infection of Small-pox after what must have been pronounced successful inoculation. The occurrence of Small-pox after Vaccination does not, we apprehend, exceed this ratio. We are aware that a fallacy in conclusion may arise out of the plainest premises. In the present instance, we go no further in our induction than our own facts warrant. The comparison with failures in Vaccination is an assumption; but we fully believe that Small-pox has not succeeded to Vaccination in the proportion of one in three thousand.

Though our observations on this short pamphlet have extended beyond the bounds usually allotted to such productions, we cannot refuse to quote its concluding paragraphs as expressive of the opinions of men whose reputations place them above the suspicion of sinister motives.

"The Board are of opinion, that Vaccination still rests upon the basis on which it was placed, by the Reports of the several Colleges of Physicians and Surgeons of the United Kingdom, which were laid before Parliament in the year 1807. That the general advantages of Vaccination are not discredited by the instances of failure which have recently occurred, the proportion of failures still remaining less in number than the deaths which take place from the Inoculated Small-pox. They are led by their information to believe, that since this practice has been fully established, no death has in any instance occurred from Small-pox after Vaccination—That in most of the Cases in which Vaccination has failed the Small-pox has been a disease remarkably mild, and of unusually short duration; and they are further of opinion, that the severity of the symptoms with which Mr. Grosvenor was affected, forms an exception to a general rule.

That absolute security from the natural Small-pox is not even to be obtained by Small-pox Inoculation, is sufficiently evident from the annexed Cases, and the board are enabled to state, that they have been made acquainted with instances of individuals who have twice undergone the natural Small-pox.

Under

Under all these circumstances, the Board feel justified in still recommending and promoting Vaccination, and in declaring their unabated confidence in this practice.—Since, in some peculiar frames of constitution, the repetition of Small-pox is neither prevented by Inoculation nor casual infection, the Board are of opinion that in such peculiar constitutions, the occurrence of Small-pox after Vaccination may be reasonably expected, and perhaps in a greater proportion, but with this admission they do not hesitate to maintain, that the proportionate advantages of Vaccination to individuals and the public, are infinitely greater than those of Small-pox Inoculation.

They are anxious that the existence of certain peculiarities of the human frame, by which some individuals are rendered by nature, more or less susceptible of eruptive fevers, and of the recurrence of such disorders, should be publicly known; for they feel confident that a due consideration of these circumstances, and a just feeling of the welfare of the Community, will induce the public to prefer a mild disease like Vaccination, which where it fails of superseding the Small-pox, yet mitigates its violence, and prevents its fatal consequences, to one whose effects are frequently violent, to one which often occasions deformity and blindness, and when it is contracted by casual infection, has been supposed to destroy one in six in all that it attacks. And it must not be forgotten, that in a public view this constitutes the great objection to Inoculation of the Small-pox, that by its contagion it disseminates death throughout the Empire, whilst Vaccination, whatever be the comparative security which it affords to individuals, occasions no subsequent disorder, and has never by the most violent of its opposers been charged with producing an epidemical sickness.

---

*A Letter respectfully addressed to the Commissioners for Transports, Sick and Wounded Seamen, &c. &c. &c. on the Subject of Operation for Popliteal Aneurism. Illustrated with Cases, and the Description of a new Instrument.* By ALEX. COPLAND HUTCHISON, M. D. Surgeon to the Royal Naval Hospital at Deal. 8vo. Callow. London. 1811. pp. 19. Plate.

THE operation for Popliteal Aneurism, as it used to be performed, was so generally followed by an unfortunate termination, that Surgeons of great judgment considered amputation of the limb to be the preferable resource. Mr. Hunter obviated much of this hazard by operating on the fore part of the thigh, and cutting down on the sound artery by the inner margin of the Sartorius muscle. Though it appears that Dominique Anel, who lived in the early part of the 18th century, had performed something like this operation in aneurism of the brachial artery, yet it is probable Mr. Hunter was the first surgeon who adopted this mode on principle; ciple; and to him the Surgeons of the present time are indebted for their success in operating for Popliteal Aneurism.

The principle was suggested by Mr. Hunter, but the operation has been improved since his time. In cutting down upon the inner margin of the Sartorius, the Vena saphena major will generally be divided, as will the principal lymphatics of the leg. Two inconveniences will arise from the division of these vessels. The flow of blood from the vein will greatly embarrass the operator, and the discharge of lymph will retard the healing of the wound. To avoid these accidents, it has been proposed to cut upon the artery by the outer margin of the Sartorius. Mr. Charles Bell first advised this method of bringing into view the femoral artery, but assigned no reason for it. Dr. Hutchison briefly states its advantages.

"There are no large veins or lymphatics in the way of the knife, and the operation will be finished in as short a time, with as little pain to the patient, and certainly with much greater satisfaction to the operator, from his not being embarrassed by hæmorrhage; a circumstance so frequently occurring, when operating on the part as directed by Mr. Hunter. In the first of the two cases here related, not more than half an ounce of blood was lost, and the greater part of that came from a minute cuticular artery. In the last there was not more in all than two drachms."

Dr. Hutchison very candidly states the objections to this method of operating.

"As the artery may be said to be nearer the inner than the outer margin of the Sartorius, and the muscle will necessarily be more disturbed in the operation when the incision is made on its outer margin; its cellular connexions with the subjacent parts will be deranged to a greater extent, and consequently the formation of larger collections of pus more favoured, and which will not have so ready an exit from the incision being less dependent."

This objection, which at first sight seems well founded, is best answered by the success of the cases related, and which we shall here quote as illustrative of the practice.

Case 1.—"Serjeant Froadsham, of the Marines, aged forty-eight, came under my care, from his Majesty's ship Bellona, on the 26th June, 1810, with a large aneurismal tumor situated on the fore part of the thigh, occupying one-third of its whole length from the inner condyle of the femur upwards, in the direction of the artery. The disease was of nearly five years standing, brought on by a long and fatiguing march to head-quarters with a deserter. According to the account given by the patient himself, he felt something snap in his thigh as he was ascending a hill, which produced considerable pain at the time, but after two or three days rest this pain subsided, and he walked about as usual—three or four days after this the pain returned, and on his examining the part, he he discovered a small pulsating tumor, not larger than the size of a hazel nut.

"On his admission into the hospital the circumference of the thigh over the aneurismal tumor was five inches greater than the opposite one at the same point; and although the integuments over it were greatly distended, there was neither inflammation or any other morbid appearance of the parts, save that of a small ecchymosed spot, the size of the point of one's finger, which did not appear to me to have any connection with the disease in question. The blood in the sac was fluid, and the pulsations of the tumor were strong—his leg and foot were slightly œdematous—he had considerable pain in the knee, and had not been able to walk for many months—he was of a very irritable habit, and had laboured under an asthmatic cough for upwards of fourteen years. His bowels were opened—℥xvi. of blood were taken from the arm, and on the 5th July the operation was performed in the following manner;

"A tourniquet being loosely applied round the upper part of his thigh, and a flannel roller passed round his foot and leg, the patient was laid upon the table in the operation-room, with the muscles, on the anterior part of the thigh, a little relaxed by means of pillows placed under the outside of the knee; an incision, nearly four inches in length, was made with one stroke of the scalpel down to the outer margin of the sartorius muscle, terminating at the commencement of the tumor: the muscle being thus exposed, was separated from its bed by the handle of the scalpel, fully half way across its width; the femoral artery became then apparent, beating in its sheath; with a pair of dissecting forceps I raised the sheath, and made a small opening into it, which was enlarged to the extent of three-fourths of an inch, by means of a probe-pointed bistoury. The artery was carefully detached from the femoral vein and saphena branch of the anterior crural nerve with my fingers and the handle of the instrument I had last used; a double ligature was then passed under the artery, with the aneurismal needle in common use, and the upper one tied as high as the vessel had been insulated; when all pulsation in the tumor at that instant ceased: in like manner the other was tied below, and the artery divided between them—both ligatures were laid out immediately opposite their respective nooses—the sides of the wound were brought in contact by the dry suture, and the thigh was surrounded with a twelve-tailed bandage, which I found to be the most convenient, as the wound could then be examined without the slightest disturbance to the position of the limb. The patient was then carried to bed—the limb placed as during the operation, and in two hours its heat was equal to that of the sound one---no numbness, pain, or irritation, succeeded to the operation; but the patient complained of a sense of trickling round the knee and throughout the whole course of the tibia; which was readily accounted for, by the blood forcing its passage through the circumflex and collateral branches, in greater quantity than they had been accustomed to carry. In the evening he was prescribed an anodyne draught, consisting of Tinct. Opii. gtt. xlv.

"2d. day---Slept five hours during the night, and no bad symptom this morning---tumor sensibly dimished, and the blood in the sac coagulated.

"4th day---His cough has been very troublesome during the last

 thirty-six thirty-six hours, accompanied with pain in the chest, slight dyspnœa, flushed countenance, and a full pulse, not exceeding ninety-five in a minute; ℥xx. of blood were therefore abstracted from the arm, and as his bowels were constipated, a dose of magnesia vitriolata was immediately directed to be taken; but which, however, proved inert till assisted by a purgative injection. The opiate was ordered to be repeated at bed time.

"Next morning (the 5th) he was free from complaint, with the exception of the trickling sensation mentioned above, which, he said, produced a slight degree of pain. This day the wound was examined, and adhesion found to have taken place throughout its whole extent, excepting where the ligatures came out: from these small openings there was rather a copious discharge of serous thin pus; some degree of tension and inflammation also surrounded the wound, but which yielded in twenty-four hours, to the constant repetition of emollient cataplasms laid over the parts every three hours; and, at the expiration of that time, the discharge was found much improved in quality.

"No other bad symptom occurred during the remainder of the cure, but the discharge of well secreted pus through the ligature-openings continued until the 14th day, when the last ligature came away. From this period until the 2d or 3d of August the discharge gradually diminished, and the wound was cicatrised on the 7th.

"The tumor continued to decrease daily, until his discharge from the hospital.—It was then barely discernable. I heard of him within the last month, when the accounts were so favourable, that, to use his own expression, there was no vestige of the tumor left, and he could then walk without the least *limp*, which, he said, he had not been able to do for years before.

"I am strongly inclined to believe that the pneumonic symptoms which immediately succeeded the operation, had been principally instrumental in favouring the extraordinary formation of matter found under the muscle during the cure. The patient's asthmatic complaint was aggravated by this attack, to such an extent, that whenever he coughed, the affected leg and thigh, with the whole frame, were so violently agitated, as to occasion great apprehensions of an hæmorrhage, by detaching the upper ligature from the extremity of the divided artery, during these vehement muscular concussions."

Case 2.—"Burnett Allan, seaman, aged thirty-two, was admitted into the hospital for Popliteal Aneurism, on the 9th November, 1810, from his Majesty's hospital ship Gorgon.

"The disease, as near as could be calculated, was then only of three months standing, and for the production of which the patient could assign no ostensible cause. When first the tumour was discovered, it had reached the size of a small walnut, and continued gradually to increase until the day of the operation, at which period it exceeded half the size of a large lemon, longitudinally and equally divided. Its pulsations were strong, but unaccompanied with pain, except when he walked—the integuments were healthy, and the leg and foot, as in the former case, were slightly œdematous—his general health was good. He was a short muscular man, of a plethoric habit of body; of a mild, patient disposition, never desponding.

"During

"During his residence in the Gorgon, the surgeon of that ship requested the opinions of the physician and surgeons of the fleet, with respect to the propriety of performing the operation on board, in the then incipient state of the disease; but these gentlemen advised the operation to be postponed, till the collateral branches should become sufficiently dilated, to ensure a due supply of blood to the limb below, when the great communicating channel should be wholly cut off.

"From the opinions of such a respectable body of my professional brethren, at that time, concurring with my own; I delayed the operation, upon the same principle, until the 19th of February following.* Six weeks previous to this, the patient was kept upon low diet. He was bled on the 18th, and his bowels were freely opened.

"The operation was then performed in the same manner as described in the foregoing case. The incision, by the outer margin of the sartorius muscle, was three inches in length, and the femoral artery was tied about an inch above where it pierces the tendon of the triceps. There was an embarrassing circumstance, however, attending this operation, which did not occur in the former, and which I think worthy of notice. After having slit open the sheath, and in detaching the artery from the vein and nerve, I discovered a perforating branch, of considerable magnitude, going off from the posterior part of the artery, exactly in the centre between where the two ligatures were to be applied, which, if the utmost caution had not been observed, (with the assistance I obtained from the finger instrument and the ivory handles of the scalpel and bistoury) the dissection might have been spoiled, by the profuse issue of blood, and the operation not completed in a desirable manner.

"The only difference occasioned by this circumstance was, that it protracted the operation somewhat longer than it otherwise would have been, and necessarily compelled me to pass the aneurismal needle twice under the artery, *viz.* above and below the perforating branch.

"It might have been advisable, perhaps, to slit open the sheath a little more downwards, so as to enable me to apply both ligatures below the perforating branch, and thereby preserved the aid of so considerable a vessel, in affording nourishment to the inferior parts of the limb; had not the femoral artery been partly insulated above the branch in question, before it was discovered. I conceive a secondary hæmorrhage, after this operation, is the grand point to be guarded against; and when it does occur, it is, in nine cases out of ten, owing to ulceration of the coats of the artery, from having its cellular connexions to the surrounding parts destroyed above the ligature, which deprives the denuded vessel of its usual supply of nourishment.

"Having now exceeded the limits which I originally proposed this paper should extend to, I shall conclude by merely acquainting the reader, that throughout the cure there did not occur one untoward symptom. There was no discharge, excepting what arose from the superficial line of the incision, until the 21st day, when the last ligature was removed, which was followed by two or three drops of pus.

---

* Had Mr. Ramsden's valuable observations on this subject sooner met my eye, I might not have delayed the operation, after the patient's admission, longer than was necessary to prepare him.

"All

"All perceptible pulsation had ceased in the aneurismal sac, from the completion of the operation; and, at the period of his dismissal from the hospital, the tumor had entirely disappeared, leaving the limb in full possession of its customary functions."

One of the objects of Dr. Hutchison's pamphlet is, to describe an instrument which is employed to retract one side of the wound; and which, occupying less space than an assistant's fingers, was found extremely convenient in a deep incised wound, at the bottom of which the surgeon has to tie the femoral artery. The most laboured description would not make our readers fully comprehend this instrument; we refer them, therefore, to the plate which accompanies the pamplet.

The value of a book is not always to be estimated by its extent. This small pamphlet clearly states practical facts of great importance; and the result of the method of operating for Popliteal Aneurism employed by Dr. Hutchison was so completely successful in the cases related, that we cannot recommend it too forcibly to the consideration of our chirurgical readers.

---

*A Posological Companion to the London Pharmacopœia. By* John Nott, M. D. of Bristol Hot-Wells; and Member of the Royal College of Physic, in London. The third edition, adapted to the last reform of the College. 18mo. pp. 109. Callow. 1811.

The object of this neat publication is to assist the young physician in the art of prescribing. "From the most respectable authorities, and from some experience, are given the doses of all the articles of the Materia Medica, and of the several medicinal preparations; the relative proportion of the principal ingredient in each preparation is also now first pointed out. Such articles and preparations, in the former, as are rejected in the present pharmacopœia, are beside comprised; some of them are excellent, and still in favour with many practitioners, notwithstanding the judicious emendations that distinguish the last labour of the College; the principal merit of which is, that of having now made the chymist and pharmaceutist to speak the same language in science with the rest of his brethren on the continent." We can safely say the author has not promised more than he has performed, and have no hesitation in recommending his little work to those who may require its aid.

